# Introducing the PRIDE Archive RESTful web services

**DOI:** 10.1093/nar/gkv382

**Published:** 2015-04-22

**Authors:** Florian Reisinger, Noemi del-Toro, Tobias Ternent, Henning Hermjakob, Juan Antonio Vizcaíno

**Affiliations:** European Molecular Biology Laboratory, European Bioinformatics Institute (EMBL-EBI), Wellcome Trust Genome Campus, Hinxton, Cambridge, CB10 1SD, UK

## Abstract

The PRIDE (PRoteomics IDEntifications) database is one of the world-leading public repositories of mass spectrometry (MS)-based proteomics data and it is a founding member of the ProteomeXchange Consortium of proteomics resources. In the original PRIDE database system, users could access data programmatically by accessing the web services provided by the PRIDE BioMart interface. New REST (REpresentational State Transfer) web services have been developed to serve the most popular functionality provided by BioMart (now discontinued due to data scalability issues) and address the data access requirements of the newly developed PRIDE Archive. Using the API (Application Programming Interface) it is now possible to programmatically query for and retrieve peptide and protein identifications, project and assay metadata and the originally submitted files. Searching and filtering is also possible by metadata information, such as sample details (e.g. species and tissues), instrumentation (mass spectrometer), keywords and other provided annotations. The PRIDE Archive web services were first made available in April 2014. The API has already been adopted by a few applications and standalone tools such as PeptideShaker, PRIDE Inspector, the Unipept web application and the Python-based BioServices package. This application is free and open to all users with no login requirement and can be accessed at http://www.ebi.ac.uk/pride/ws/archive/.

## INTRODUCTION

Mass spectrometry (MS)-based proteomics analysis techniques are increasingly used in the life sciences. The PRIDE (PRoteomics IDEntifications) database (1) (http://www.ebi.ac.uk/pride) at the European Bioinformatics Institute (EBI) is one of the world-leading public repositories for storing MS-based proteomics data. PRIDE stores, among other data types, peptide and protein identifications and related quantification values, the corresponding mass spectra (both as processed peak lists and raw data) and any other technical and/or biological metadata provided by the submitters. PRIDE is leading the ProteomeXchange Consortium (2) (http://www.proteomexchange.org) of MS proteomics resources, which aims to standardize data submission and dissemination of this type of data worldwide. Within the Consortium, PRIDE fully supports the storage of tandem MS data (by far the main approach used in the field today), although data coming from other proteomics workflows can be also stored (e.g. top down proteomics or data independent acquisition approaches). It is important to note that, unlike other resources that reanalyse the data using their own data analysis pipelines, PRIDE stores all result data types as originally analysed by the authors. The implementation of ProteomeXchange has resulted in a big increase in public data deposition in the field. By March 2015, around 1900 datasets had been submitted to the ProteomeXchange resources since mid 2012, when the data workflow within the Consortium was formalised. Of those, more than 90% were stored in PRIDE.

The PRIDE project started around 10 years ago (3). However, the original system was built to support smaller-scale experiments, and its infrastructure could no longer be maintained with the rise of new high-throughput workflows, producing ever-growing file volumes and new data types. The new PRIDE Archive system has now been developed from scratch following the ProteomeXchange guidelines and supporting the Proteomics Standard Initiative (PSI) community data standards mzML (4), mzIdentML (5) and mzTab (6), although other data formats (e.g. mgf, mzXML, raw files, etc.) are also supported (7). At present there are different ways to access this plethora of data: the PRIDE web interface, the file repository (which supports the FTP and Aspera (http://asperasoft.com/) file transfer protocols) and the stand-alone PRIDE Inspector tool (8). Until December 2014, it was possible to access PRIDE data programmatically by accessing the RESTful (service based on REpresentational State Transfer protocol) web services provided by the PRIDE BioMart interface (9). However, the BioMart interface was recently discontinued due to the lack of product support and increasing scalability issues. New RESTful web services have been developed to replace the most popular functionality available in the BioMart and to serve the data access requirements of the newly developed PRIDE Archive. Among the other major public proteomics data resources, GPMDB (10) has also REST-style web services available (11) (http://rest.thegpm.org/1) whereas PeptideAtlas (12) does not provide this functionality.

In this manuscript we describe the main features of these new web services, which can be freely accessed at http://www.ebi.ac.uk/pride/ws/archive/.

## ARCHITECTURE, DESIGN AND IMPLEMENTATION

To ensure maintainability and adequate support, the web services have been developed using technologies that are also used across other PRIDE projects. The services are implemented in Java, building on top of the Spring framework (http://projects.spring.io/spring-framework/). Data queries are powered by optimized Apache Solr servers (http://lucene.apache.org/solr/). Data can be accessed over HTTP (HyperText Transfer Protocol) via REST-like ‘Get’ requests, which ensures that the services are easy to use and are supported by all major platforms. JSON (JavaScript Object Notation) was chosen as the output format since it is widely used as a data serialization format and is the current *de facto* data exchange standard for web technologies.

### Cross Origin request

When the web service is used from within other web applications, the Same Origin Policy (SOP, which is implemented in all browsers for security reasons), is in effect. This may prevent the application to directly access the data in PRIDE. Traditionally such sites had to implement a local proxy to get around the restrictions of the SOP. However, in recent years other ways to make possible Cross Origin Requests have emerged. The web service currently supports two of the most common ones:

(i) JSONP (JSON with Padding): JSONP callbacks are supported with the optional request parameter ‘callback’.
(ii) CORS (Cross-Origin Resource Sharing): the web services are configured to add cross-origin allow headers to all responses.

For further details, consult the documentation pages (http://www.ebi.ac.uk/pride/help/archive/access/webservice).

## DATA SEARCH AND RETRIEVAL

The PRIDE Archive Web Service API is split into several specific resources, which currently are projects, assays, files, protein identifications and peptide identifications. This separation is also reflected in the service URLs (Uniform Resource Locator), where the first level after the web service root determines the resource or data type. Data retrieval options depend on the information available at each level.

Specific project and assay records can be retrieved using their respective accession numbers as available via the PRIDE Archive web interface and the ProteomeXchange portal ProteomeCentral (http://proteomecentral.proteomexchange.org/cgi/GetDataset). Projects (which correspond to individual dataset submissions) can also be queried by their associated metadata, identified proteins (using their accession numbers) and peptides (using their amino acid sequence). Among others, metadata includes annotations for the samples (such as species, tissues, detected post-translational modifications, diseases, etc.) and other experimental details that are gathered during the data submission.

**Figure 1.**
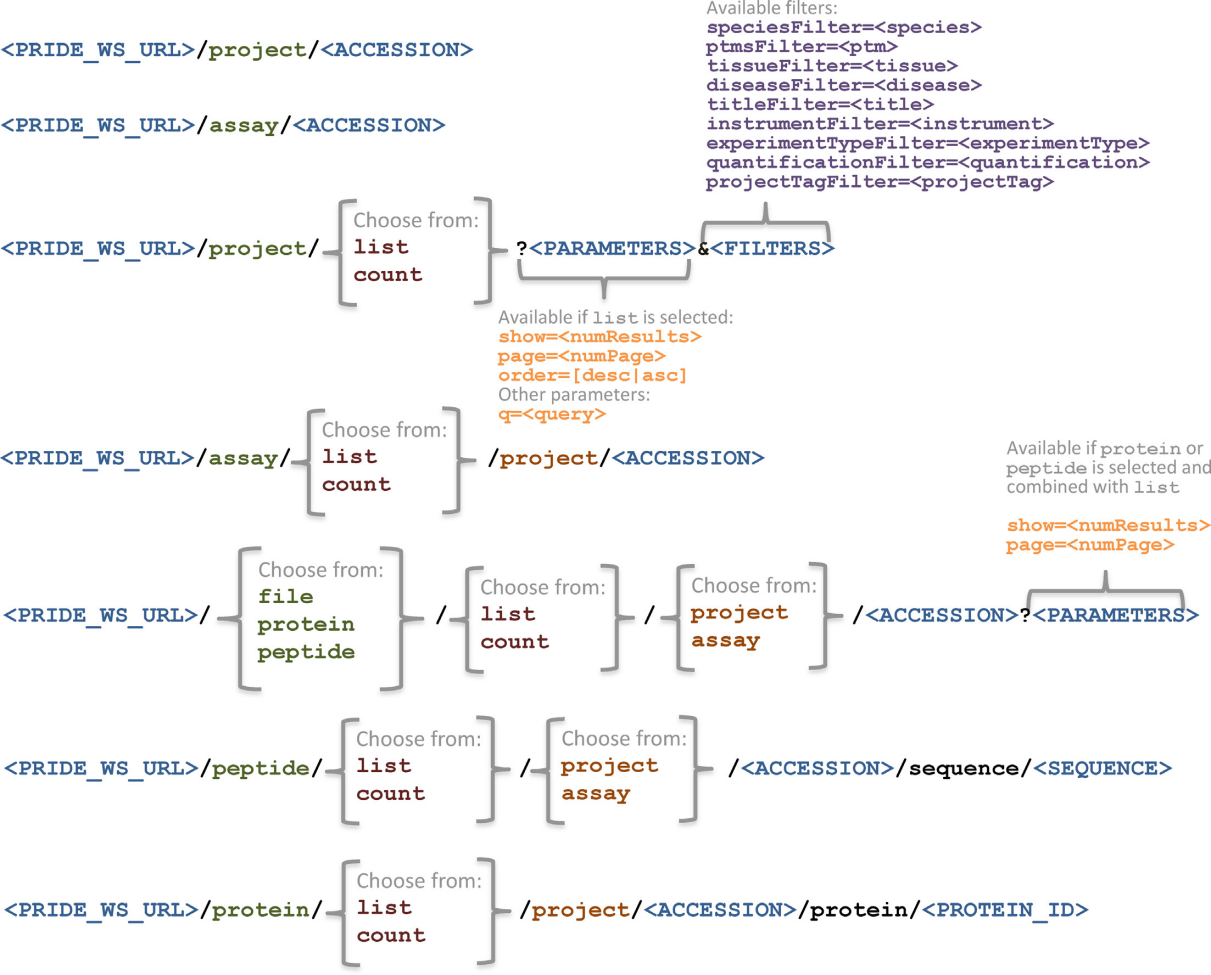
Structure of resources and methods available for the clients to fetch data from the PRIDE Archive RESTful web services.

Once a project of interest has been identified, other web service methods enable the retrieval of specific records, such as protein and peptide identifications and a list of URLs to download the originally submitted files. Then, users can combine web service functionalities to achieve more complex queries/results, for example the retrieval across projects of peptide/protein identifications, as described in the online documentation (see http://www.ebi.ac.uk/pride/help/archive/access/webservice). The detailed list of current methods is available at Table 1. In an attempt to make the service more intuitive and easier to use, several conventions were introduced (Figure 1):

(i) as already mentioned, the first level after the web service root (http://www.ebi.ac.uk/pride/ws/archive) denotes the type of resource requested (project, assay, file, protein or peptide).
(ii) if the end-point URL contains the keyword ‘list’, then a list of entities is returned, rather than a single entity.
(iii) similarly, if the URL contains the keyword ‘count’ an integer number is returned, showing the total of entities that would be returned by the equivalent ‘list’ method. This is particularly useful when dealing with paged results (see below).

**Table 1.** Currently supported PRIDE Archive web service methods

Resource	URLs (to go after http://www.ebi.ac.uk/pride/ws/archive)	Description
Project	/project/{projectAccession}	Retrieve project information by accession
	/project/list	List projects for given criteria
	/project/count	Count projects for given criteria
Assay	/assay/{assayAccession}	Retrieve assay information by accession
	/assay/list/project/{projectAccession}	List assays for a project
	/assay/count/project/{projectAccession}	Count assays for a project
File	/file/list/project/{projectAccession}	List files for a project
	/file/count/project/{projectAccession}	Count files for a project
	/file/list/assay/{assayAccession}	List files for an assay
	/file/count/assay/{assayAccession}	Count files for an assay
Protein	/protein/list/project/{projectAccession}	Retrieve protein identifications by project accession
	/protein/count/project/{projectAccession}	Count protein identifications by project accession
	/protein/list/project/{projectAccession}/protein/{protein}	Retrieve protein identifications by project accession and protein accession
	/protein/count/project/{projectAccession}/protein/{protein}	Count protein identifications by project accession and protein accession
	/protein/list/assay/{assayAccession}	Retrieve protein identifications by assay accession
	/protein/count/assay/{assayAccession}	Count protein identifications by assay accession
Peptide	/peptide/list/project/{projectAccession}	Retrieve peptide identifications by project accession
	/peptide/count/project/{projectAccession}	Count peptide identifications by project accession
	/peptide/list/project/{projectAccession}/sequence/{sequence}	Retrieve peptide identifications by project accession and peptide sequence
	/peptide/count/project/{projectAccession}/sequence/{sequence}	Count peptide identifications by project accession and peptide sequence
	/peptide/list/assay/{assayAccession}	Retrieve peptide identifications by assay accession
	/peptide/count/assay/{assayAccession}	Count peptide identifications by assay accession
	/peptide/list/assay/{assayAccession}/sequence/{sequence}	Retrieve peptide identifications by assay accession and peptide sequence
	/peptide/count/assay/{assayAccession}/sequence/{sequence}	Count peptide identifications by assay accession and peptide sequence

### Paging and sorting

Certain end-points make use of paging and sorting to enable a more efficient access to the data (see Figure 1 and the online documentation for details). Methods that make use of paging have a corresponding count method, so users can check the total number of results before deciding how or if paging is necessary. Paging is essential to guarantee reasonable response times when result sets grow very large, which is often the case for data coming from high-throughput proteomics experiments. It is also crucial to improve the responsiveness in client applications and free the client from having to deal with large data volumes if perhaps only a preview is desired, or if the client is too small to process the full data load at once (e.g. for mobile devices). The service allows results to be ordered according to certain criteria, which is often needed along with the paging. For instance, sorting criteria for project lists include the publication date, its accession number or title, and the relevance score assigned to each result.

### Querying and filtering

The web service therefore offers query functionality that is closely modelled on the search of the PRIDE Archive web interface. For a list of the available filters, their descriptions and examples, see Table 2. Users can search the repository using generic query terms, restrict results by applying designated filters or use a combination of both. To illustrate the difference, consider the following example. If a *diseaseFilter* with the value ‘cancer’ is used, only projects that carry ‘cancer’ as disease annotation are retrieved. However, if ‘cancer’ is used as generic query term, the result of the query will also contain projects with an annotation which mentions this keyword, but may not specifically in the disease-type annotation.

**Table 2.** Summary of the supported values as filters for the queries

Available filters	Description	Ontology or control vocabulary (CV)	Examples
speciesFilter	Species name or taxonomy identifier	NCBI taxon ID	9606 /pride/ws/archive/project/list?speciesFilter = 9606
ptmsFilter	Protein modification name	Unimod or PSI-MOD	phosphorylation /pride/ws/archive/project/list?ptmsFilter = phosphorylation
tissueFilter	Tissue name	BRENDA or Experimental Factor Ontology (EFO)	brain/pride/ws/archive/project/list?tissueFilter = brain
diseaseFilter	Disease name	Human Disease Ontology (DOID) or EFO	cancer/pride/ws/archive/project/list?diseaseFilter = cancer
titleFilter	Words included in the title of the project	Free text	stress/pride/ws/archive/project/list?titleFilter = stress
instrumentFilter	MS instrument name	PRIDE or PSI-MS	ltq/pride/ws/archive/project/list?instrumentFilter = ltq
experimentTypeFilter	Experiment type name	PRIDE or PSI-MS	shotgun/pride/ws/archive/project/list?experimentTypeFilter = shotgun
quantificationFilter	Quantification method name	PRIDE or PSI-MS	lable-free /pride/ws/archive/project/list?quantificationFilter = label-free
projectTagFilter	Keywords added by PRIDE curators to describe the project	Examples of tags: ‘Biological’, ‘Biomedical’, ‘Technical’, ‘Cardiovascular’, ‘Highlighted’. For more details visit http://www.ebi.ac.uk/pride/help/archive/tags	Biomedical /pride/ws/archive/project/list?projectTagFilter = Biomedical

### Example use case

A user of the web services might be interested in retrieving identification data for projects related to ‘biomarkers for human cancer’. Since metadata annotations, like biomarkers, tissue or disease related information are available on project level and not in the individual protein or peptide identification level, the first step would be to find relevant projects.

(i) A request to /pride/ws/archive/project/list?query = biomarkers will produce a list of projects that contain annotations including the word ‘biomarkers’. By applying additional disease and species filters …?query = biomarkers&diseaseFilter = cancer&speciesFilter = 9606, the result can be further restricted.
(ii) Examining the details of the result, a project of interest can be identified. To retrieve the full record for a specific project, for example PXD001034, a user would send a request to /pride/ws/archive/project/PXD001034.
(iii) To then retrieve all protein identifications for that project, a user could first use /pride/ws/archive/protein/count/project/PXD001034 to find out how many protein identifications are expected (see the section devoted to paging above).
(iv) With requests to /pride/ws/archive/protein/list/project/PXD001034 a user would then retrieve the lists of the protein identification records.
(v) Similarly, in order to retrieve the original project files, the web services can be queried to provide a list of file records containing the URLs for all the files included in a given project: /pride/ws/archive/file/list/project/PXD001034. A user can then inspect the file records and decide to download all the files or only those of a particular type.

Other example use cases can be found in the web services online documentation (see http://www.ebi.ac.uk/pride/help/archive/access/webservice).

## API DOCUMENTATION

**Figure 2.**
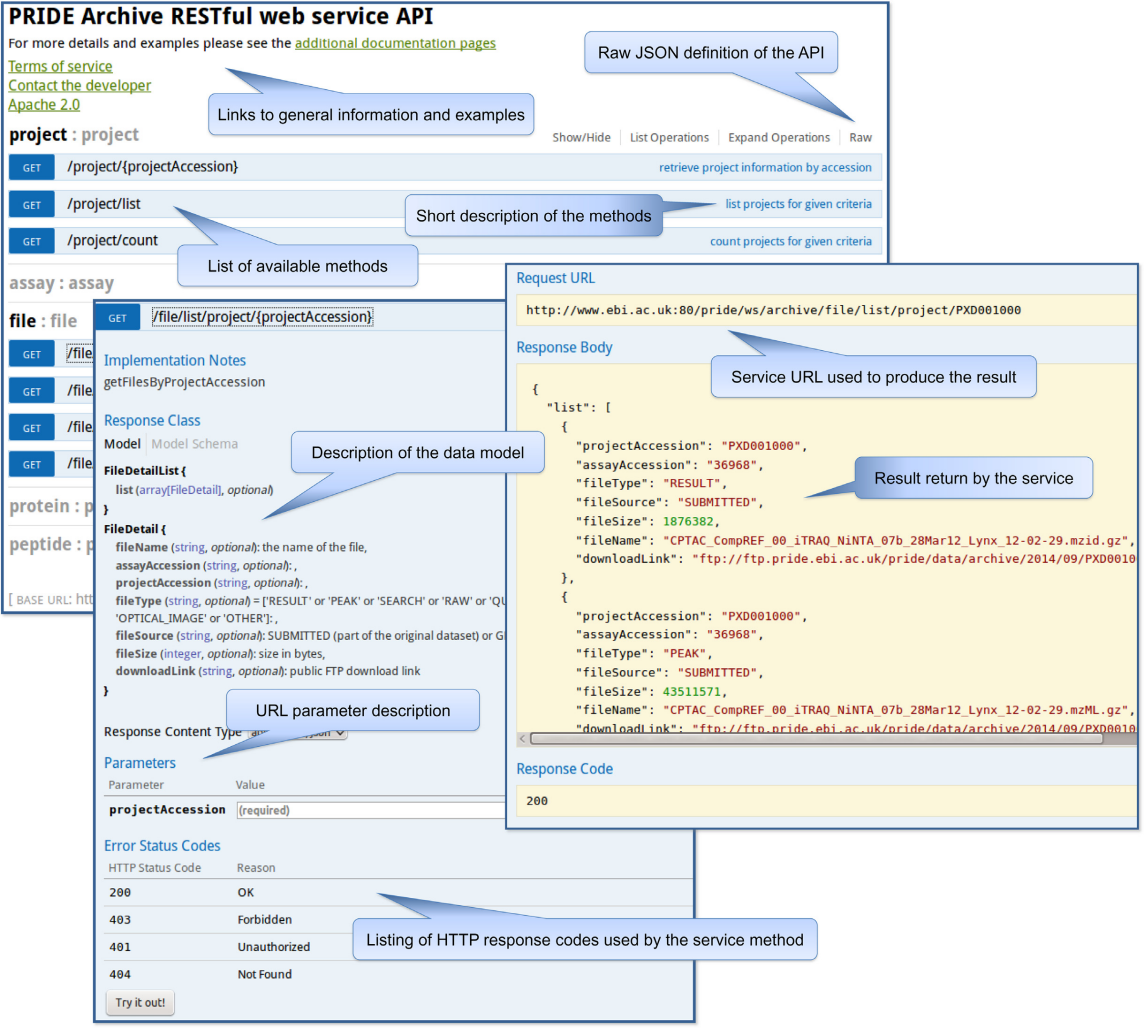
Schema of the interactive documentation of the PRIDE Archive web services implemented using the Swagger™ framework.

The available documentation is divided in two parts. First of all, using the popular documentation framework Swagger™ (http://swagger.io/), an interactive auto-generated documentation is available in the home page (http://www.ebi.ac.uk/pride/ws/archive/). The goal of Swagger™ is to define a standard, language-agnostic interface to REST APIs which allows both humans and computers to discover and understand the capabilities of the service without access to source code, documentation or through network traffic inspection (https://github.com/swagger-api/swagger-ui). It lists all the available end-points and provides definitions and descriptions of the methods, parameters and the data model (Figure 2). The interactive part allows the execution of example queries using simple input forms. The results are rendered in the same page to allow a quick examination. The interface also shows the URL that would have to be used by a client in order to perform the same request. As this documentation is auto-generated from the source code, it is always up-to-date with the latest release.

In addition to the auto-generated documentation, further information is available in the general PRIDE ‘Help’ pages. These contain, among others, descriptions of general concepts, example use cases and other content that is not covered by the Swagger™ framework. The documentation also contain links to example clients in Python and Java that can be used as starting points or templates for developers wishing to use the web services (see http://www.ebi.ac.uk/pride/help/archive/access/webservice).

## DISCUSSION

The PRIDE Archive RESTful web services have been developed to enable programmatic access to PRIDE Archive data. This is a key development for users due to the ever-increasing data volume that PRIDE is experiencing and the fact that data mining, data reanalysis and reinterpretation are currently flourishing in the proteomics field (13,14). The API has been available since April 2014 and a few internal and external applications are already making use of the new functionality: (i) the stand-alone tool PeptideShaker (https://code.google.com/p/peptide-shaker/) (15), which is an analysis tool that, among other functionality, enables the reanalysis of PRIDE data accessing PRIDE files and metadata using the ‘PRIDE Reshake’ option; (ii) the PRIDE Inspector, a visualization and validation tool developed by the PRIDE team. Using the API, PRIDE Inspector enables a generic project search and the access to any project in PRIDE Archive, including private datasets (protected by an username and password). This way, PRIDE Inspector can be used by journal reviewers and editors during the manuscript review process; (iii) the UniPept web application (http://unipept.ugent.be/) (16), a metaproteomics resource, which makes use of the API to access peptide sequence information; and (iv) the Python-based BioServices package (17), which can be used to access several major bioinformatics resources including now PRIDE (http://pythonhosted.org//bioservices/references.html#module-bioservices.pride).

The PRIDE REST web services functionality will continue to develop in parallel with the PRIDE Archive. Should users wish to discuss requests for new functionality, the authors encourage them to contact the PRIDE helpdesk (pride-support@ebi.ac.uk) with their suggestions.
